# Ronald J. Burke and Lisa M. Calvano (eds): The Sandwich Generation. Caring for Oneself and Others at Home and at Work: Edward Elgar

**DOI:** 10.1007/s10680-017-9449-x

**Published:** 2017-11-22

**Authors:** Anita Abramowska-Kmon

**Affiliations:** Demography Unit, Institute of Statistics and Demography, Warsaw School of Economics, Warsaw, Poland

The book entitled “The Sandwich Generation. Caring for Oneself and Others at Home and at Work” (edited by Ronald J. Burke, Lisa M. Calvano) examines the exchange of care between generations and its impact on caregivers’ lives from different theoretical and practical perspectives. Moreover, it addresses the important issue of supporting carers in their responsibilities towards family members and professional work by organizations and social policies.

The volume is composed of 12 chapters, which are organized in four parts. Almost all chapters (with the exception of two) are based on the review of existing theories and empirical studies. The first part “Introduction and context” focuses on a general situation of the sandwich generation, and more broadly the caregivers, and the different aspects of caregiving and their effects on people’s lives and their daily functioning. In particular, the introductory chapter is devoted to the multifaced issue of caregiving. It also presents the challenges caregiving creates for individuals, families, organizations and societies. The next chapter which is an interesting example of empirical analyses, investigates different aspects of caregiving and its impact on, among others, caregivers’ lives, employment and quality of life of sandwiched caregivers in comparison with other caregivers. The last chapter of this part presents socio-demographic perspectives on intergenerational relations in later life which shape care exchanges between generations. The authors discuss various aspects of intergenerational relations and their impact on relatives’ readiness and willingness to help those in need.

Part II “Taking care of caregivers” is composed of two chapters. The first one addresses the issue of supporting the caregiver of a person with dementia disease. The second one is a review of theoretical considerations and empirical research on resources alleviating the negative consequences of combing work and engagement in some form of caregiving (of children, older persons, or both).

Part III entitled “The important role of organizations” brings up the issue how caregivers function at work, while they are involved in supporting other people. Chapter 6 is a review of the research results on objective and perceived support caregivers receive from organizations/employers. The section focuses solely on persons caring for children or older persons. The section also indicates the future research directions which would be beneficial in widening the knowledge on interrelation between caregiving, employment, organizational support and well-being of carers. Chapter 7 addresses similar issues but from the perspective of the sandwich generation only. In particular, it concentrates on policies and organizational practices aiming at supporting those providing care to two generations at the same time in order to increase their well-being.

The last part (IV “Policy context”) covers several issues related to care in diverse policy contexts. For example, chapter 8 describes how different policy contexts (cultural and structural factors) influence the provision of work-life policies by organizations as well as needs, expectations and use of the support policies by employed caregivers. Chapter 9 concentrates on health inequalities between older Americans of different ethnic groups, in particular showing disadvantages among African Americans in comparison with other racial groups. Although this chapter raises many important issues related to health inequalities and situation of African Americans in the US, it seems to be unrelated to the remaining parts of the volume (at least directly). Chapter 10 presents results of empirical analyses dedicated to the relationship between mature women’s involvement in caregiving and their employment in the Australian older people’s care services. This is a nice mixture of quantitative and qualitative work, which raises the attractiveness of the chapter. In Chapter 11 a description of childcare and eldercare policies in Sweden is given. Part IV and the book ends with a short chapter on personal experiences of one of the editors (Lisa M. Calvano) in caring for the dependent mother.

The book focuses on different types of care provided by persons aged 45–65 (to children or older persons in need) and only a part of it is strictly dedicated to the sandwich generation, i.e. those in-between the care needs of younger and older generations. Moreover, it deals with the difficulties experienced by caregivers resulting from a simultaneous involvement in work and in caregiving as well as its impact on well-being of carers. This approach seems to be appropriate because research suggests that caretaking of children and parents at the same time is rather a rare phenomenon. What seems to pose a challenge for caregivers is thus not the fact of being “squeezed” between generations, but rather the difficulties with combining care responsibilities with paid work. This difficulty is not to be experienced by a specific generation only, but it rather refers to a situation which will be a part of life of almost all of us, but at different ages and to a different extent. Thus, in this context, the title of the book is a bit misleading, suggesting that the main focus will be on the sandwich generation.

Nevertheless, the volume is a valuable source of information on many issues related to the caregiving from the perspective of theoretical considerations, empirical research and practical policy making. It can thus be of interest for researchers, practitioners and all people involved in supporting others, as it shows what kind of strategies and actions should be undertaken by policy makers and individuals in order to support caregivers. This is especially crucial in light of ongoing population ageing and the growing number of older people who require help; a process which will put more pressure on potential caregivers. These caregivers may be at various stages of the life course and take care of a dependent child, elderly parents or a spouse. This may result in possibly different needs when it comes to support from other family members or public care sector.

